# Screening asymptomatic men for prostate cancer: A comparison of international guidelines on prostate-specific antigen testing

**DOI:** 10.1177/09691413221119238

**Published:** 2022-09-04

**Authors:** Sherena D Jackson, May R de la Rue, Thomas PL Greenslade, Anna M John, Shahida Wahid, Richard M Martin, Naomi J Williams, Emma L Turner

**Affiliations:** Bristol Medical School, 1980 University of Bristol, Canynge Hall, 39 Whatley Road, Bristol, BS8 2PS, UK

**Keywords:** Prostate-specific antigen (PSA), prostate cancer, cancer screening, guidelines, international comparison

## Abstract

**Objective:**

To summarise and compare the key recommendations on prostate-specific antigen (PSA)-based screening for prostate cancer, and so highlight where more evidence is required to facilitate consistent recommendations.

**Methods:**

The Medline database and websites of 18 national screening organisations and professional associations were searched between January 2010 and November 2020 to identify screening guidelines published in English, considering recent clinical trials.

**Results:**

Population-based PSA testing of asymptomatic men is not widely recommended. Guidelines emphasize shared patient-clinician decision making. For ‘average-risk’ men choosing to be screened, the recommended age varies from 50–55 to 70 years, alongside consideration of life expectancy (ranging from 7–15 years). Screening intervals, when specified, are biennial (most common), annual, or determined from baseline PSA. The earliest age for screening high-risk men (frequently defined as of African descent or with a family history of prostate cancer) is 40 years, but recommendations often defer to clinical judgement.

**Conclusions:**

Population screening of asymptomatic men is not widely recommended. Instead, balancing the potential harms and benefits of PSA testing is endorsed. Variation between guidelines stems from differing interpretations of key trials and could lead to clinician-dependent screening views. The development of clinical decision aids and international consensus on guidelines may help reduce national and international variation on how men are counselled.

## Introduction

Prostate cancer (PCa) is the second commonest malignancy and the fifth leading cause of cancer death in men worldwide. The early detection and prompt treatment of potentially life-threatening PCa is key to any policy aimed at reducing mortality. However, screening asymptomatic men using the serum prostate-specific antigen (PSA) test is a contentious public health issue. Although the PSA test itself is useful in monitoring PCa progression, it lacks specificity in the detection of clinically important PCa in asymptomatic men.^1^ The identification of indolent cancers introduces the potential for harm, either from complications arising from unnecessary investigations such as infection after biopsy,^2^ or from treatment complications such as incontinence or impotence after surgery or radiotherapy (‘over-treatment’).^3^ False-negative PSA tests may offer false reassurance, leading to delayed presentation with more advanced disease and a poorer prognosis.^1^

Three randomized controlled trials (RCTs) investigated the effect of PSA-based screening on PCa mortality: the Prostate, Lung, Colorectal and Ovarian Cancer Screening [PLCO] trial of annual PSA screening (N  =  76,693); the European Randomized Study of Screening for Prostate Cancer [ERSPC] involving 2–4 yearly screening (N  =  162,243); and the Cluster Randomized Trial of PSA Testing for Prostate Cancer [CAP] involving a single PSA screen (N  =  408,825). ERSPC showed a reduction in PCa mortality among men randomized to four-yearly screening (rate ratio: 0.80 [95% CI: 0.72 to 0.89]) at 16 years follow-up.^4^ In PLCO at 13 years follow-up, annual screening increased PCa diagnoses (rate ratio: 1.12 [95% CI: 1.07 to 1.17]), but without evidence of a PCa mortality reduction (rate ratio: 1.09 [95% CI  =  0.87 to 1.36).^5^ CAP showed no difference in PCa mortality between men from general practices randomized to a single screen and men receiving standard care at a median 10 years follow-up (rate ratio: 0.96 [95% CI: 0.85 to 1.08]), but an increase in the detection of low-risk PCas among the screened group (rate ratio: 1.19 [95% CI: 1.14 to 1.25]).^1^ There is, therefore, uncertainty about the magnitude of the effects of PSA-based screening on PCa mortality and whether any potential benefits outweigh the harms from over-detection or overtreatment, leading to different guidelines on PSA screening worldwide.

This paper summarises and compares the key recommendations on PSA-based screening for PCa for both average-risk (someone with no identifiable PCa risk factors) and high-risk (those of African descent or with a family history of PCa) men, identifying areas of disagreement. Our overarching objective was to highlight areas where more evidence is required to facilitate consistent PSA screening recommendations.

## Methods

Medline was searched using the terms listed in Appendix 1 (online supplementary material). Records were exported to be screened in Rayyan.^6^ We conducted a grey literature search of websites associated with national screening and professional organisations. References to unidentified guidelines were used to uncover any that were missed (snowball sampling).

Two assessors, among five reviewers overall [MdLR, TG, SJ, AJ, SW], screened each record against inclusion crietria and differences resolved by consensus, as illustrated in a PRISMA flow diagram (Figure 1, Appendix 2 in the online supplementary material). Information was abstracted on the main recommendations for different target populations, stratified by age, risk status and screening intervals.

## Results

Published recommendations on PSA testing for asymptomatic men were identified for nine countries or regions, and 18 national or professional organisations, as shown in Table 1. The references and comprehensive recommendations are available in Appendix 3 (online supplementary material).

**Table 1.** Guidelines for men at average and high risk.

Country	Organisation	Year	Recommendations for average risk men	Recommendations for high risk men
Australia	Prostate Cancer Foundation of Australia and Cancer Council Australia	2015	Biennal screening if PSA<3 ng/mL for ages 50-59 and LE >7	Test biennially (PSA dependent) if discussion had for ages 40-69 years No further testing until 50 if PSA ≤75^th^ percentile for age
Canada	Canadian Task Force on Preventive Health Care	2014	Recommends against screening for ages 55–70	No specific recommendation, encourage discussion Risk factors- Family history, men of black race
	Canadian Urological Association	2017	Offer PSA testing following discussion from ages 50-70 and LE >10 Test every 4 years if PSA <1 ng/mL Biennial if PSA 1-3 ng/mL More frequent if PSA >3 ng/mL	Offer PSA testing following discussion Risk factors- Family history, men of black race
Denmark	Danish Urological (Prostate) Cancer Group (DAPROCA)	2019	Recommends against both systematic and opportunistic screening for all ages	PSA testing can be offered to men >45 years and LE >10-15 Risk factor- Family history
Europe	EAU-EANM-ESTRO-ESUR-SIOG^a^	2020	Offer an individualised risk-adapted strategy to well informed men if >50 years and LE >10-15 Biennial follow-up if PSA >1ng/mL at 40years or >2ng/mL at 60 years	Offer early testing if >40 years and LE >10-15 years Risk factors- Family history, African descent
	European Society for Medical Oncology (ESMO)	2020	Offer early PSA testing followed by risk adapted strategy if >50 years and LE >10	Offer early testing if >40 years and LE >10 years Risk factors- Family history, African-American, genetics
Ireland	National Cancer Control Programme	2018	Recommends shared decision making if <50–70 years. PSA level dependent biennial PSA testing	No specific recommendations Risk factors- Family history, African descent
Japan	Japanese Urological Association	2016	Population-based screening if >50 years Test every 3 years if PSA <1 ng/mL. Annually if PSA meets age-specific cut-offs	No specific recommendations Risk factors- Family history, genetic mutations
New Zealand	Prostate Cancer Working Group & Ministry of Health	2015	Test every 2-4 years if discussion has been had to those ages 50-70	Test annually for men ages 40-70 following discussion Risk factors- Family history
UK	UK National Screening Committee	2020	Recommends against systematic population screening for all ages	No specific recommendations Risk factors- Black ethnicity, family history age, genetics, higher BMI
	Prostate Cancer Risk Management Programme (PCRMP)	2020	PSA testing available on request for ages >50 GP should not proactively raise issue	Use clinical judgement when offering testing to those <50 Risk factors- Family history, men of black race
	International panel (The BMJ Rapid Recommendations)^b^	2018	Recommends shared decision-making for all ages	No specific recommendations Risk factors- Family history, African descent, low socioeconomic status
United States	US Preventive Services Task Force (USPSTF)	2018	Recommends shared decision making if 55-69 years of age. Against screening if >70	No specific recommendation, but inform men if ages 55-69 years of age Risk factors- Family history, African-American
	American Cancer Society	2020	Recommends shared decision making. For ages >50 and LE >10 years, test biennially if PSA <2.5 ng/mL, annually if >2.5 ng/mL	Informed decision making if ages >40 and LE >10 years Risk factors- Family history, African-American
	American Urological Association	2018	Recommends shared decision making. Biennial screening if 55-69 years	Informed decision making for ages 40-54 Risk factors- Family history, African-American
	American Academy of Family Physicians	2020	Recommends shared decision making. Biennial screening for ages 55-69	No specific recommendations but inform men if ages 55-69 Risk factors- Family history, African-American
	American College of Physicians	2013	Recommends shared decision making for ages 50-69 years	Recommends shared decision making for men aged >40 Risk factors- Family history, African-American
	National Comprehensive Cancer Network (NCCN)	2019	Recommends shared decision making. Test every 2-4 years if PSA<1 ng/mL or 1-2 years if PSA 1-3 ng/mL for ages 45-75 years	Recommends shared decision making and annual PSA testing if >40 years Risk factors- genetics, African-American

Note: Comprehensive recommendations and references are available in Appendix 3 (Tables 1 and 2, online supplementary material).

PSA: prostate-specific antigen; LE: life expectancy.

^a^ European Associaion of Urology – European Association of Nuclear Medicine – European Society for Radiology and Oncology – Uropean Society of Urogenital Radiology– International Society of Geriatric Oncology.

^b^ A panel of men at risk of prostate cancer, general practitioners, general internists, urologists, epidemiologists, methodologists, and statisticians.

Table 1 summarises the key recommendations for men at ‘average and high risk’ of PCa. There is general agreement against recommending ‘population-based’, ‘systematic’ or ‘routine screening’ for prostate cancer. The Japanese Urological Society (JUA) is the only organisation to specifically recommend a ‘population-based screening’ programme in men over 50 years.

Guidelines converge on the importance of shared decision making between men and their clinicians, but many lack clarity on who should lead the discussion. The UK’s Prostate Cancer Risk Management Programme (PCRMP) recommends that clinicians should not proactively raise the issue with average-risk men and should exercise “clinical judgement” in the case of high-risk men under 50 years. For average-risk men, most guidelines recommend 50 or 55 years as an appropriate age to start the discussion and to cease around 70 years. The exceptions are the National Comprehensive Cancer Network (NCCN), which recommends that screening discussions occur between the ages of 45 and 75 years, and the National Cancer Control Programme (NCCP), which recommends screening discussions for men younger than 50 years.

Most guidelines recommend a life expectancy of at least 10 years when considering screening, except the Prostate Cancer Foundation of Australia and Cancer Council Australia (PCFACCA) who recommend 7 years for average-risk men. How to assess life expectancy objectively is unclear.

There is less guidance on testing frequency. Ten of the organisations addressed recommend screening biennially, whilst others suggest annual and some recommendations are based on a baseline PSA level.

Six guidelines provide ‘no specific recommendations’ for those considered high risk but some of these suggest it would be reasonable to have a shared discussion. The critera for ‘high risk’ vary (most specifiy family history and being of Black race or African-American descent). A lower screening discussion age of 40 or 45 years, if specified, is recommended.

## Discussion

There is widespread rejection of population-based screening using the PSA test and an emphasis on providing opportunities for shared decision making. This shift towards more patient-centred care is arguably needed in this setting where the potential for harm is significant.^2^

The ambiguity about whether clinicians introduce the discussion could lead to subjective screening decisions and impact the number of individuals counselled.^7^ If the man is responsible, influences of their education level and health literacy may lead to health inequities.^8^

Certain factors, such as African descent and family history, increase an individual’s PCa risk. Yet only 5% of participants enrolled in the PLCO trial were Black men and the ERSPC trial did not analyse this demographic, despite Black men being twice as likely to die from PCa as Caucasian men.^9^ Some organisations do not provide specific recommendations for high-risk men leading to poor clarity and subjectivity. Non-uniformity in medical funding and access to healthcare may play a role in magnifying disparities particularly amongst high-risk men.

Research in PSA screening could consider both increasing the recruitment of high-risk men into clinical trials, and focus on the clinician’s role in raising PCa screening. The generation of decision aids may illustrate and aid existing evidence as well as improve knowledge and reduce decision conflict amongst patients and clinicians.^10^ These advances could engender more cohesion among recommendations, with a shift towards more evidence-based policy.

## Supplemental Material

sj-docx-1-msc-10.1177_09691413221119238 - Supplemental material for Screening asymptomatic men for prostate cancer: A comparison of international guidelines on prostate-specific antigen testing Supplemental material, sj-docx-1-msc-10.1177_09691413221119238 for Screening asymptomatic men for prostate cancer: A comparison of international guidelines on prostate-specific antigen testing by Sherena D Jackson, May R de la Rue, Thomas PL Greenslade, Anna M John, Shahida Wahid, Richard M Martin, Naomi J Williams and Emma L Turner in Journal of Medical Screening
