# Supplementary material for: Screening asymptomatic men for prostate cancer: A comparison of international guidelines on prostate-specific antigen testing
Source: J Med Screen. 2022 Sep 4;29(4):268–71. doi: 10.1177/09691413221119238 (PMC9574423; doi:10.1177/09691413221119238)
Supplement: sj-docx-1-msc-10.1177_09691413221119238 - Supplemental material for Screening asymptomatic men for prostate cancer: A comparison of international guidelines on prostate-specific antigen testing [file sj-docx-1-msc-10.1177_09691413221119238.docx]

**APPENDIX 1**

(prostate[Title] OR prostate cancer[Title] OR prostate specific antigen[Title] OR prostate-specific antigen[Title] OR PSA[Title]) AND (screening[Title] OR screen[Title] OR guidance[Title] OR guideline[Title] OR guidelines[Title] OR recommendation[Title] OR recommendations[Title]) AND (UK[Title/Abstract] OR United Kingdom[Title/Abstract] OR US[Title/Abstract] OR United States[Title/Abstract] USA[Title/Abstract] OR Europe[Title/Abstract] OR European[Title/Abstract] OR Luxembourg[Title/Abstract] OR Switzerland[Title/Abstract] OR Ireland[Title/Abstract] OR Norway[Title/Abstract] OR Denmark[Title/Abstract] OR Netherlands[Title/Abstract] OR Iceland[Title/Abstract] OR Austria[Title/Abstract] OR Sweden[Title/Abstract] OR Germany[Title/Abstract] OR Belgium[Title/Abstract] OR Australia[Title/Abstract] OR Finland[Title/Abstract] OR Canada[Title/Abstract] OR France[Title/Abstract] OR New Zealand[Title/Abstract] OR Korea[Title/Abstract] OR Japan[Title/Abstract] OR American[Title/Abstract] OR Swiss[Title/Abstract] OR Canadian[Title/Abstract] OR Irish[Title/Abstract] OR Norwegian[Title/Abstract] OR Danish[Title/Abstract] OR Holland[Title/Abstract] OR Austrian[Title/Abstract] OR Swedish[Title/Abstract] OR German[Title/Abstract] OR Belgian[Title/Abstract] OR Australian[Title/Abstract] OR Finnish[Title/Abstract] OR French[Title/Abstract] OR Korean[Title/Abstract] OR Japanese[Title/Abstract] OR Dutch[Title/Abstract]

**APPENDIX 2**

**Figure 1: PRISMA flow diagram ^35^**

**Identification of studies via other methods**

**Identification of studies via databases and registers**

Records identified from Medline database (n = 620)

Records identified from:

Organisation websites (n=41)

Records removed *before screening*:

Duplicate records removed (n = 0)

Records marked as ineligible by automation tools (not in English) (n =48 )

**Identification**

Records screened

(n = 572 )

Records excluded

(n = 494)

Records sought for retrieval

(n =41)

Records not retrieved

(n =5)*

Records sought for retrieval

(n = 78)

Records not retrieved

(n =1 )

**Screening**

Records excluded:

Not an organisation guideline (n = 57)

Not a top 20 OECD country (n = 1)

Guideline is not regarding PCa screening (n = 9)

A more recent guideline is available (n=5)

A subset of a guideline (n=1)

Records assessed for eligibility

(n = 36)

Records excluded:

Previously identified in database search (n=4)

More recent guidelines available (n=3)

Not original guidelines (n=14)

Records assessed for eligibility

(n = 77)

Guidelines included in review

(n = 19)

**Included**

Records were included if they were:

1. the most recent prostate cancer screening guidelines issued by an organisation;
2. published by organisations within countries ranked in the top 20 OECD grouping;
3. published in the English language;

between January 2010 and November 2020 were included as these dates are post publication of the first results of ERSPC and PLCO.

*Belgium, Iceland, Finland, Luxembourg, and the Netherlands.

**APPENDIX 3**

**Table 1: Guidelines for men at average risk**

| **Country** | **Organisation** | **Year** | **Ages and life expectancy (years)** | **Recommendations for men at Average risk** | **Screening intervals** |
| --- | --- | --- | --- | --- | --- |
| Australia | Prostate Cancer Foundation of Australia and Cancer Council Australia^11^ | 2015 | All ages | Recommends against population-based screening | - |
|  |  |  | 50-69 & LE >7 | Offer PSA testing to men who have been informed of the benefits and harms of testing. Provide evidence-based decisional support to men considering screening | Biennial if PSA <3ng/mL |
| Canada | Canadian Task Force on Preventive Health Care^12^ | 2014 | <55 and >70 | Recommends against screening. Clinicians should not routinely discuss screening for prostate cancer | - |
|  |  |  | 55-69 | Recommends against screening. Clinicians should discuss the risks and benefits of screening and its potential consequences with each man in the context of his preferences |  |
|  | Canadian Urological Association^13^ | 2017 | 50-70 & LE >10 | Offer PSA testing. The decision should be based on shared decision-making after the potential benefits and harms have been discussed | Every 4 yrs if PSA <1 ng/mL  (discontinue if PSA <1 ng/mL at >60 yrs)  Biennial if PSA 1-3 ng/mL  More frequent testing if PSA >3 ng/mL |
|  |  |  | >70 & LE >10 | Recommends discontinuation of PSA testing; however, for interested men in excellent health at age 70, continued PSA testing can be considered depending on clinical judgment and personal preferences |  |
| Denmark | Danish Urological (Prostate) Cancer Group (DAPROCA)^14^ | 2019 | All ages | Recommends against both systematic and opportunistic screening | - |
| Europe | ^(^^[[1]](#footnote-1))^EAU-EANM-ESTRO-ESUR-SIOG^15^ | 2020 | >50 & LE > 10-15 | Offer an individualised risk-adapted strategy for early detection to a well-informed man with a good performance status. Do not subject men to PSA testing without counselling them on the potential risks and benefits | Risk-adapted strategy (based on initial PSA) with biennial follow-up for those initially at risk: (PSA >1 ng/mL at 40 yrs,  PSA >2 ng/mL at 60 yrs)  Postpone to 8 yrs in those not at risk |
|  | European Society for Medical Oncology (ESMO)^16^ | 2020 | All ages | Recommends against population-based screening |  |
|  |  |  | >50 & LE >10 | Offer early PSA testing (baseline PSA, followed by risk-adapted follow-up) |  |
| Ireland | National Cancer Control Programme^17^ | 2018 | All ages | PSA should not be considered a routine test and informed consent should be obtained | - |
|  |  |  | <50 and 50-70 | If PSA is being considered, recommends shared decision-making. GP should discuss patient’s concerns, benefits/harms/risks of prostate assessments and provide patient information | Biennial if PSA:  <2 ug/mL at <50 yrs  <3 ng/ml at 50-59 yrs  <4 ng/mL at 60-69 yrs |
|  |  |  | >70 & LE >10 | For men who are healthy and fit, recommends shared decision-making. GP should discuss patient’s concerns, benefits/harms/risks of prostate assessments and provide patient information. Recommends against testing in men >70 with life-limiting comorbidities | - |
| Japan | Japanese Urological Association^18^ | 2016 | >50 | Population-based screening: screen those who have received an explanation of the latest information as well as the benefits and drawbacks and have granted consent.  The decision to continue screening in the elderly is challenging because of the difficulty of accurately predicting life expectancy. Consider the use of Geriatric health assessment screening tools | Every 3 yrs if baseline PSA ≤1.0 ng/mL.  Annual if PSA is between 1.1 ng/mL and age-specific cut-offs:  3.9 ng/mL at 50-64 yrs  3.5 ng/mL at 65-69 yrs  4.0 ng/mL at ≥70 yrs |
| New Zealand | Prostate Cancer Working Group & Ministry of Health^19^ | 2015 | 50-70 | Primary care practitioners should discuss the benefits and risks of screening | Every 2-4 years, depending on personal preference |
| UK | UK National Screening Committee^20^ | 2020 | All ages | Recommends against systematic population screening | - |
|  | Prostate Cancer Risk Management Programme (PCRMP)^21^ | 2020 | >50 | PSA testing available on request. PCRMP information available to help GPs give clear and balanced information to men who ask about PSA testing however GPs should not proactively raise the issue of PSA testing with asymptomatic men | - |
|  | ^(^^[[2]](#footnote-2))^International panel (The BMJ Rapid Recommendations)^22^ | 2018 | All ages | Recommends against systematic PSA screening | - |
|  |  |  | All ages | Recommends shared decision-making for all men considering screening to make a decision consistent with their individual values and preferences. However, clinicians need not feel obligated to systematically raise the issue of PSA screening with their patients |  |
| United States | US Preventive Services Task Force (USPSTF)^23^ | 2018 | 55-69 | The decision to be screened should be an individual one. Men should have an opportunity to discuss the potential benefits and harms of screening with their clinician and to incorporate their values and preferences in the decision. Clinicians should not screen men who do not express a preference for screening | - |
|  |  |  | >70 | Recommends against PSA-based screening for prostate cancer |  |
|  | American Cancer Society^24^ | 2020 | >50 & LE >10 | Recommends that men should have a chance to make an informed decision with their health care provider about whether to be screened. The decision should be made after getting information about the uncertainties, risks and potential benefits of screening. Men should not be screened unless they have received this information | Biennial if PSA <2.5 ng/mL  Annual if PSA ≥2.5 ng/mL |
|  | American Urological Association^25^ | 2018 | <40 | Recommends against PSA screening | - |
|  |  |  | 40 – 54 | Does not recommend routine screening. Decisions about screening should be individualised | Biennial (or less frequent; ≥4 yrs if PSA <1 ng/mL at age >60)  Re-screening intervals can be individualised based on baseline PSA |
|  |  |  | 55-69 | Recommends shared decision-making for men considering PSA screening, and proceeding based on a man’s values and preferences |  |
|  |  |  | >70 or LE <10-15 | Does not recommend routine screening. However some men aged over 70 who are in excellent health may benefit from prostate cancer screening |  |
|  | American Academy of Family Physicians^26^ | 2020 | 55-69 | Does not recommend routine PSA screening. For men who are considering screening, clinicians should discuss the risks and benefits and engage in shared decision-making that enables an informed choice | Biennial |
|  |  |  | >70 | Recommends against screening for prostate cancer | - |
|  | American College of Physicians^27^ | 2013 | <50, >69 or LE <10 | Clinicians should not screen for prostate cancer | - |
|  |  |  | 50-69 | Recommends that clinicians inform men about the limited potential benefits and substantial harms of screening for prostate cancer. The decision to screen should be based on the risk for prostate cancer, a discussion of the benefits and harms of screening, the patient’s general health and life expectancy and patients preferences. Only men who express a clear preference for screening should be screened |  |
|  | National Comprehensive Cancer Network (NCCN)^28^ | 2019 | 45-75 | Discuss the risks and benefits of screening before making an informed, shared decision (Baseline PSA) | Every 2-4 yrs if PSA <1 ng/mL  Every 1-2 yrs if PSA 1-3 ng/mL |
|  |  |  | >75 | Recommends against widespread screening in this age group. Testing should only be done in very healthy men with little or no comorbidity (especially if they have never undergone PSA testing) |  |

**Table 2: Guidelines for men at high risk**

| **Country** | **Organisation** | **Year** | **Ages, Life expectancy (years) & specified risk factors** | **Recommendations for men at High risk** | **Screening intervals** |
| --- | --- | --- | --- | --- | --- |
| Australia | Prostate Cancer Foundation of Australia and Cancer Council Australia^11^ | 2015 | 40-69: & LE >7: more than one 1^st^ degree relative  45-69 & LE>7: 1^st^ degree relative (particularly if <60 at diagnosis) | Offer PSA testing for men younger than 50 years who are concerned about their risk for prostate cancer and have been informed of the benefits and harms of testing | No further testing until 50 if PSA ≤75^th^ percentile for age.  Biennial if PSA >75^th^ but <95^th^ percentile for age |
| Canada | Canadian Task Force on Preventive Health Care^12^ | 2014 | Family history, men of black race | No specific recommendations - however, clinicians may wish to discuss the benefits and harms of screening in men at higher risk, with explicit consideration of their values and preferences | - |
|  | Canadian Urological Association^13^ | 2017 | 45-70 & LE>10: Family history, black men | Offer PSA testing. The decision should be based on shared decision-making after the potential benefits and harms have been discussed | - |
|  |  |  | BRCA1/BRCA2/HOXB13 | Suggest an individualized testing strategy after consultation with a clinical geneticist |  |
| Denmark | Danish Urological (Prostate) Cancer Group (DAPROCA)^14^ | 2019 | >45 & LE>10-15: Family history | ‘The PSA test can be offered to the well-informed, family-disposed male’ | - |
| Europe | ^(^^[[3]](#footnote-3))^EAU-EANM-ESTRO-ESUR-SIOG^15^ | 2020 | >40 & LE >10-15: BRCA2  >45 & LE >10-15: Family history, African descent | Offer early PSA testing to well-informed men | - |
|  | European Society for Medical Oncology (ESMO)^16^ | 2020 | >40 & LE>10: BRCA1/2  >45 & LE>10: Family history, African-American | Offer early PSA testing (baseline PSA, followed by risk-adapted follow-up) | *-* |
| Ireland | National Cancer Control Programme^17^ | 2018 | African ethnicity, Family history & early age of onset (<50), BRCA1/2 | No specific recommendations | - |
| Japan | Japanese Urological Association^18,29^ | (2010) 2016 | >40: Family history, genetic mutations (HOXB13 G84E) | No specific recommendations | - |
| New Zealand | Prostate Cancer Working Group & Ministry of Health^19^ | 2015 | 40-70: Family history (at least one 1^st^ degree relative & early age of onset) | Primary care practitioners should discuss the benefits and risks of testing.  Considerations for men considering PSA testing include, ethnicity (Maori) and demographics (residence in rural or low-decile communities). However, no specific recommendations given for these groups of men | Annual |
| UK | UK National Screening Committee^20^ | 2020 | Black ethnicity, Family history Age, BRCA1/2, Higher BMI, Exposure to pesticides | No specific recommendations | - |
|  | Prostate Cancer Risk Management Programme (PCRMP)^21^ | 2020 | <50: Black ethnicity, Family history | GPs should use their clinical judgement to manage those aged under 50 who are considered to be at higher risk of prostate cancer | - |
|  | ^(^^[[4]](#footnote-4))^International panel (The BMJ Rapid Recommendations^22^ | 2018 | African descent , 1^st^ degree relative, Lower socioeconomic status | No specific recommendations | - |
| United States | US Preventive Services Task Force (USPSTF)^23^ | 2018 | 55-69: Family history, African American | No specific recommendation due to lack of evidence – however, a reasonable approach for clinicians is to inform these groups about their increased risk and discuss the potential benefits and harms of screening so they can make in informed, personal decision about whether to be screened | - |
|  |  |  | >70: Family history, African American | Recommends against PSA-based screening for prostate cancer |  |
|  | American Cancer Society^24^ | 2020 | **>40 & LE>10: multiple** 1^st^ degree relatives with PCa at an early age  **>45 & LE >10:** African American, 1^st^ degree relative with PCa at an early age (<65) | Recommends that men should have a chance to make an informed decision with their health care provider about whether to be screened. The decision should be made after getting information about the uncertainties, risks and potential benefits of screening. Men should not be screened unless they have received this information | - |
|  | American Urological Association^25^ | 2018 | **40-54: African American, Family history of metastatic or lethal adenocarcinomas in multiple generations, affecting multiple 1st degree relatives & developed at younger ages** | Decisions about screening should be individualised. These men should be informed of both the known harms and the potential benefits of screening at an earlier age and shared decision-making should ensue with an understanding that there are no comparative data to demonstrate than men at higher than average risk for prostate cancer will benefit more from screening when compared to those at average risk | - |
|  | American Academy of Family Physicians^26^ | 2020 | 55-69: African American, Family history | No specific recommendation due to lack of evidence – however, these men should be informed of their increased risk of developing prostate cancer in addition to the benefits and harms of screening so that they can make an informed choice | - |
|  | American College of Physicians^27^ | 2013 | >40: multiple family members diagnosed before age 65  >45: African American race, 1^st^ degree relative (especially if diagnosed <65) | Men at high risk should receive information about the uncertainties, risks, and potential benefits associated with prostate cancer screening. The importance of shared decision making in making screening choices is emphasized | - |
|  | National Comprehensive Cancer Network (NCCN)^28^ | 2019 | >40: African American, BRCA1/2 | It is reasonable for men at high risk to undergo informed, shared decision-making at an earlier age. Referral to a cancer genetics professional if there is a known or suspected cancer susceptibility gene | Annual |

**References**

1. Prostate Cancer Foundation of Australia and Cancer Council Australia PSA Testing Guidelines Expert Advisory Panel. Clinical practice guidelines PSA Testing and Early Management of Test-Detected Prostate Cancer, <https://wiki.cancer.org.au/australia/Guidelines:PSA_Testing/Summary_of_recommendations>; 2015 [Accessed 24 June 2020].
2. Canadian Task Force. Prostate Cancer. Summary of recommendations for clinicians and policy makers, <https://canadiantaskforce.ca/guidelines/published-guidelines/prostate-cancer/>; 2014 [Accessed 24 June 2020].
3. Rendon, RA, Mason, RJ, Marzouk, K, et al. Canadian Urological Association recommendations on prostate cancer screening and early diagnosis. *Canadian Urological Association Journal* 2017; 11(10): 298-309. <https://doi.org/10.5489/cuaj.4888.>
4. Danish Prostate Cancer Group. Screening & tidlig detektion af prostatacancer, <https://ducg.dk/fileadmin/ingen_mappe_valgt/5_1_PSA_og_screening_ver._1.1.pdf>; 2019 [accessed 24 June 2020].
5. EAU-EANM-ESTRO-ESUR-SIOG. Guidelines on Prostate Cancer, <https://uroweb.org/wp-content/uploads/EAU-EANM-ESTRO-ESUR-SIOG-Guidelines-on-Prostate-Cancer-2020v4-1.pdf>; 2020 [accessed 24 June 2020].
6. Parker C, Castro E, Fizazi K, Heidenreich A, Ost P, Procopio G et al. Prostate cancer: ESMO Clinical Practice Guidelines for diagnosis, treatment and follow-up. *Ann Oncol* 2020;31:1119-1134. <https://doi.org/10.1016/j.annonc.2020.06.011>.
7. Health Service Executive and National Cancer Control Programme. National prostate cancer GP referral guideline, <https://www.hse.ie/eng/services/list/5/cancer/profinfo/resources/gpreferrals/nccp-prostate-cancer-gp-referral-guideline.pdf>; 2018 [accessed 24 June 2020].
8. Kakehi Y, Sugimoto M and Taoka R. Evidenced-based clinical practice guideline for prostate cancer (summary: Japanese Urological Association, 2016 edition). *Int J Urol* 2017;24:648-666. <https://doi.org/10.1111/iju.13380>.
9. Prostate Cancer Working Group and Ministry of Health. Prostate cancer management and referral guidance, <https://www.health.govt.nz/publication/prostate-cancer-management-and-referral-guidance>; 2015 [accessed 24 June 2020].
10. UK National Screening Committee. Adult screening programme recommendation for prostate cancer, <https://legacyscreening.phe.org.uk/prostatecancer>; 2016 [accessed 24 June 2020].
11. The Prostate Cancer Risk Management Programme. Prostate specific antigen testing: summary guidance for GPs, <https://www.gov.uk/government/publications/prostate-specific-antigen-testing-explanation-and-implementation>; 2020 [accessed 24 June 2020].
12. Tikkinen KAO, Dahm P, Lytvyn L, Heen AF, Vernooij RWM, Siemieniuk RAC et al. Prostate cancer screening with prostate-specific antigen (PSA) test: a clinical practice guideline. *BMJ*. 2018;362 :k3581. https://doi.org/10.1136/bmj.k3581.
13. US Preventive Services Task Force. Screening for Prostate Cancer: US Preventive Services Task Force Recommendation Statement. *JAMA* 2018;319:1901-1913. <https://doi.org/10.1001/jama.2018.3710>.
14. American Cancer Society. American Cancer Society recommendations for prostate cancer early detection, <https://www.cancer.org/cancer/prostate-cancer/detection-diagnosis-staging/acs-recommendations.html>; 2020 [accessed 24 June 2020].
15. Carter HB, Albertsen PC, Barry MJ, Etzioni R, Freedland SJ, Greene KL et al. Early Detection of Prostate Cancer: AUA Guideline*. J. Urol* 2013;190:419. http://doi.org/10.1016/j.juro.2013.04.119.
16. American Academy of Family Physicians. Clinical preventive service recommendation for prostate cancer, <https://www.aafp.org/family-physician/patient-care/clinical-recommendations/all-clinical-recommendations/prostate-cancer.html>; 2020 [accessed 24 June 2020].
17. Qaseem A, Barry MJ. Denberg TD, Owens DK, Shekelle P, Clinical Guidelines Committee of the American College of Physicians. Screening for Prostate Cancer: A Guidance Statement from the Clinical Guidelines Committee of the American College of Physicians. *Ann Intern Med* 2013;158(10):761-769. https://doi.org/10.7326/0003-4819-158-10-201305210-00633.
18. National Comprehensive Cancer Network. NCCN Guidelines Version 2.2019: Prostate Cancer Early Detection, <https://www.nccn.org/professionals/physician_gls/pdf/prostate_detection.pdf>; 2019 [accessed 24 June 2020].
19. Committee for Establishment of the Guidelines on Screening for Prostate Cancer; Japanese Urological Association. Updated Japanese Urological Association Guidelines on prostate-specific antigen-based screening for prostate cancer in 2010. *Int J Urol* 2010;17(10):830-8. <https://doi.org/10.1111/j.1442-2042.2010.02613.x>.

1. ^()^ European Associaion of Urology – European Association of Nuclear Medicine – European SocieTy for Radiology and Oncology – Uropean Society of Urogenital Radiology– International Society of Geriatric Oncology [↑](#footnote-ref-1)
2. ^()^ A panel of men at risk of prostate cancer, general practitioners, general internists, urologists, epidemiologists, methodologists, and statisticians. [↑](#footnote-ref-2)
3. ^()^ European Associaion of Urology – European Association of Nuclear Medicine – European SocieTy for Radiology and Oncology – Uropean Society of Urogenital Radiology – International Society of Geriatric Oncology [↑](#footnote-ref-3)
4. ^()^ A panel of men at risk of prostate cancer, general practitioners, general internists, urologists, epidemiologists, methodologists, and statisticians. [↑](#footnote-ref-4)
